# The Atypical Cannabinoid Abn-CBD Reduces Inflammation and Protects Liver, Pancreas, and Adipose Tissue in a Mouse Model of Prediabetes and Non-alcoholic Fatty Liver Disease

**DOI:** 10.3389/fendo.2020.00103

**Published:** 2020-03-06

**Authors:** Silvana Y. Romero-Zerbo, María García-Fernández, Vanesa Espinosa-Jiménez, Macarena Pozo-Morales, Alejandro Escamilla-Sánchez, Lourdes Sánchez-Salido, Estrella Lara, Nadia Cobo-Vuilleumier, Alex Rafacho, Gabriel Olveira, Gemma Rojo-Martínez, Benoit R. Gauthier, Isabel González-Mariscal, Francisco J. Bermúdez-Silva

**Affiliations:** ^1^UGC Endocrinología y Nutrición, Instituto de Investigación Biomédica de Málaga-IBIMA, Hospital Regional de Málaga, Universidad de Málaga, Málaga, Spain; ^2^Departamento de Fisiología Humana, Facultad de Medicina, Instituto de Investigación Biomédica de Málaga-IBIMA, Universidad de Málaga, Málaga, Spain; ^3^Plataforma de Microscopía, Instituto de Investigación Biomédica de Málaga-IBIMA, Málaga, Spain; ^4^Andalusian Center for Molecular Biology and Regenerative Medicine (CABIMER), Seville, Spain; ^5^Laboratory of Investigation in Chronic Diseases - LIDoC, Department of Physiological Sciences, Center of Biological Sciences, Federal University of Santa Catarina (UFSC), Florianópolis, Brazil; ^6^Centro de Investigación Biomédica en Red de Diabetes y Enfermedades Metabólicas Asociadas (CIBERDEM), Madrid, Spain

**Keywords:** cannabinoids, inflammation, prediabetes, NAFLD, obesity, liver, islets of Langerhans, adipose tissue

## Abstract

**Background and Aims:** The synthetic atypical cannabinoid Abn-CBD, a cannabidiol (CBD) derivative, has been recently shown to modulate the immune system in different organs, but its impact in obesity-related meta-inflammation remains unstudied. We investigated the effects of Abn-CBD on metabolic and inflammatory parameters utilizing a diet-induced obese (DIO) mouse model of prediabetes and non-alcoholic fatty liver disease (NAFLD).

**Materials and Methods:** Ten-week-old C57Bl/6J mice were fed a high-fat diet for 15 weeks, following a 2-week treatment of daily intraperitoneal injections with Abn-CBD or vehicle. At week 15 mice were obese, prediabetic and developed NAFLD. Body weight and glucose homeostasis were monitored. Mice were euthanized and blood, liver, adipose tissue and pancreas were collected and processed for metabolic and inflammatory analysis.

**Results:** Body weight and triglycerides profiles in blood and liver were comparable between vehicle- and Abn-CBD-treated DIO mice. However, treatment with Abn-CBD reduced hyperinsulinemia and markers of systemic low-grade inflammation in plasma and fat, also promoting white adipose tissue browning. Pancreatic islets from Abn-CBD-treated mice showed lower apoptosis, inflammation and oxidative stress than vehicle-treated DIO mice, and beta cell proliferation was induced. Furthermore, Abn-CBD lowered hepatic fibrosis, inflammation and macrophage infiltration in the liver when compared to vehicle-treated DIO mice. Importantly, the balance between hepatocyte proliferation and apoptosis was improved in Abn-CBD-treated compared to vehicle-treated DIO mice.

**Conclusions:** These results suggest that Abn-CBD exerts beneficial immunomodulatory actions in the liver, pancreas and adipose tissue of DIO prediabetic mice with NAFLD, thus protecting tissues. Therefore, Abn-CBD and related compounds could represent novel pharmacological strategies for managing obesity-related metabolic disorders.

## Introduction

Western diet and sedentary lifestyle increase prevalence of obesity worldwide. Obesity is a risk factor for developing diabetes and cardiovascular and liver diseases among others, reducing disease-free years as obesity becomes more severe (1). Obesity-related disorders arise progressively; prediabetes and non-alcoholic fatty liver disease (NAFLD) are early stages of pancreatic and liver damage. Although these pathologies can be diagnosed, there are no treatments at the level of the underlying molecular mechanisms. Current biomedical research focuses on the study of the early stages of obesity-related diseases for the generation of treatments aimed at stopping its progression and complications.

It is becoming evident that dysfunction of the molecular integration of the immune and metabolic systems underlies metabolic diseases such as type 2 diabetes (2). In fact, low-grade chronic inflammation (meta-inflammation) that occurs in obesity is considered an important factor in many disorders related to obesity, including type 2 diabetes (3). Several studies have detected changes in inflammatory cytokines in people with prediabetes (4–6). The adipose tissue has been determined to be the source of a plethora of inflammatory signals that, once in circulation, induce activation of lymphocytes. Indeed, accumulation of triglycerides in non-adipose tissues triggers inflammation, macrophages infiltration and apoptosis. Sustained high blood glucose level is associated with an increase in oxidative stress and intracellular inflammation that ultimately leads to loss of beta cell mass (7, 8). In the liver, accumulation of triglycerides leads to NAFLD that eventually ends in liver failure (9).

Cannabinoids are known to modulate the metabolism of lipids and glucose as well as inflammatory processes (10). In fact rimonabant, a cannabinoid type 1 receptor (CB1R) antagonist, has been in the market for the treatment of complicated obesity, although central side effects finally led to its withdrawal (11). Since then, the development of a second-generation family of cannabinoid-based drugs without side effects for treating metabolic diseases has been the focus of intense research. Atypical cannabinoids are ligands that do not target the canonical cannabinoid receptors CB1R and CB2R. The prototype of this kind of molecules is the phytocannabinoid cannabidiol (CBD) that lacks psychoactive effects and is one of the main components of *Cannabis sativa* plant. Most synthetic atypical cannabinoids derive ultimately from CBD and include abnormal CBD (Abn-CBD), O-1602, O-1918, and O-1821 (12). CBD and some synthetic atypical cannabinoids have been reported to display anti-inflammatory and anti-oxidant properties, including potential anti-diabetic actions (13–16). Unfortunately, clinical trials with CBD have failed to demonstrate improvements in glycemic and lipid parameters in patients with type 2 diabetes (17). However, effects of synthetic atypical cannabinoids on obesity-related inflammation and early stages of related diseases remain largely unexplored.

Abn-CBD results from the transposition of the phenolic hydroxyl group and the pentyl side chain of CBD (18). *In vitro* findings point to Abn-CBD displaying modulatory actions on neutrophils in inflammatory conditions such as experimental colitis and atherogenesis (19, 20). Indeed, Abn-CBD was found to improve glucose tolerance in streptozotocin-induced diabetic mice (15). In agreement with these findings we recently reported that Abn-CBD decreases cytokine-induced apoptosis in mouse and human isolated islets while promoting beta cell proliferation (21). However, the impact of Abn-CBD on obesity-related meta-inflammation and its relationship with prediabetes and NAFLD remains unstudied.

Here we aimed to investigate the metabolic effects and the anti-inflammatory properties of Abn-CBD in the liver and pancreas of a mouse model of diet-induced prediabetes and NAFLD.

## Materials and Methods

### Study Design and Generation of Diet-Induced Prediabetic and NAFLD Mice

**Figure 1:**
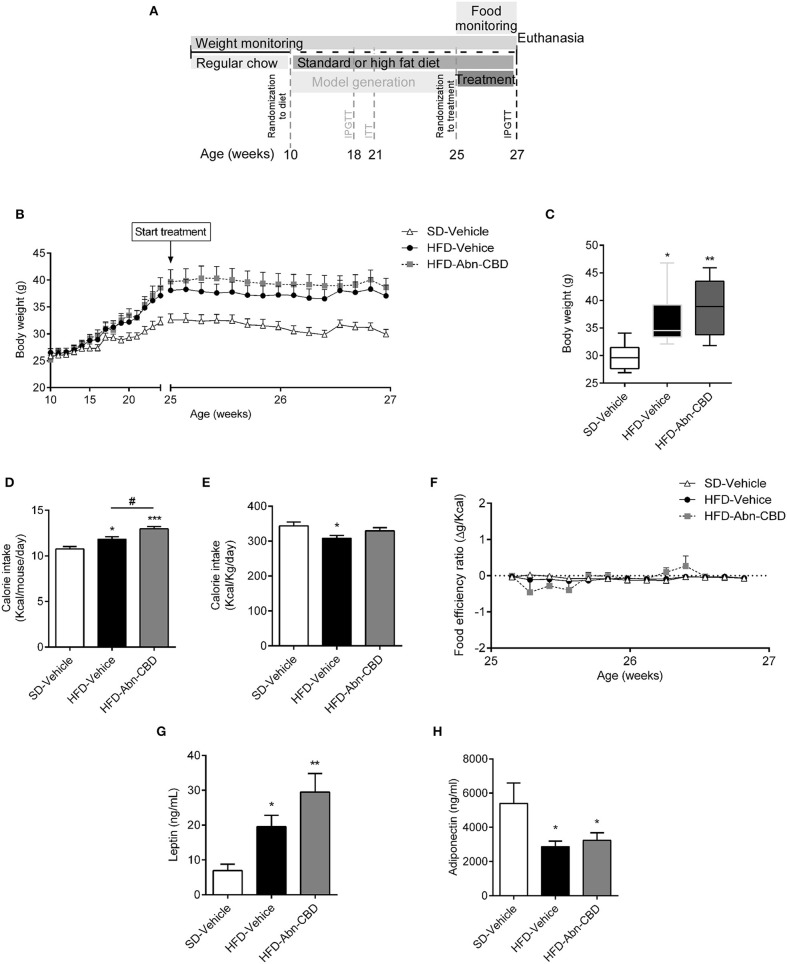
Effect of Abn-CBD on body weight and food intake. Mice fed a standard diet (SD) or high fat diet (HFD) for 15 weeks were randomized to vehicle or Abn-CBD for further 2 weeks **(A)**. Body weight was measured weekly during the first 15 weeks and daily during treatment **(B)**. **(C)** Body weight after the 2-week treatment with vehicle or Abn-CBD. **(D)** Average of daily calorie intake per mouse and **(E)** per Kg of weight during the 2-week treatment and **(F)** food efficiency. Leptin **(G)** and adiponectin **(H)** plasma levels at the end of the study. Data show mean ± S.E.M except **(C)** that shows median ± min to max. *n* = 6 SD-Vehicle, *n* = 7 HFD-Vehicle and *n* = 8 HFD-Abn-CBD. ^*^p ≤ 0.05, ^**^p ≤ 0.01, and ^***^p ≤ 0.001 compared to SD-Vehicle; ^#^*p* ≤ 0.05 compared to HFD-Vehicle.

The European Union recommendations (2010/63/EU) on animal experimentation were followed. All animal experimentations were approved by the Ethic Committee of the University of Malaga (authorization no. 2012–0061A), and followed the 3R's principle. Ten-week-old C57BL/6J male mice were purchased from Charles River (France) and were acclimatized to the animal facility for one week with food and water available *ad libitum* and lights on between 8:00 and 20:00 h. Mice were then fed a 10% fat diet (control) or a 45% fat diet (45% of Kcal from lard, saturated fats, HFD) for 15 weeks (*n* = 10 and 30 mice, respectively). Body weight was monitored twice a week and glucose and insulin tolerance assessed by intraperitoneal glucose tolerance (GTT) and insulin tolerance test (ITT), respectively. Briefly, GTT was performed after overnight fasting by injecting 2 g/kg D(+)glucose (Sigma-Aldrich, St. Louis, MO). Blood glucose was monitored from the tail vein at baseline and 15, 30, 60, and 90 min using a glucose meter (Accu-check, Roche Diagnostic). For the ITT, mice were fasted for 6 hours and then injected with 0.5 U/kg of insulin (Humulin, Lilly, France). Blood samples were collected from the tail vein as above and blood glucose was measured at the same time points with the glucometer. Glucose and insulin area under the curve (AUC) was calculated from their corresponding graphs using IMAGEJ software (National Institutes of Health, Bethesda, MA, USA). Once HFD-fed mice showed greater body weight than control mice, glucose intolerance and insulin resistance, animals were randomized to vehicle or treatments and 10 mice were treated with the synthetic cannabinoid Abn-CBD. The herein study with Abn-CBD represents a subset of a larger study that also included the cannabinoid ligand LH-21. The pre-diabetic phenotype of these mice (HFD-vehicle vs. SD-vehicle) has been previously described (22). A detailed scheme of Abn-CBD study design is depicted in Figure 1A.

### Subchronic Treatment With Abn-CBD and Monitoring of Body Weight and Food Intake

HFD mice were treated daily for the last 2 weeks of the experimental design with 0.05 mg/kg of Abn-CBD (Cayman Chemical, Ann Harbor, MI) or vehicle. Dosage was selected based on previous literature (15, 23). The stock solution of Abn-CBD was dissolved in ethanol, aliquoted, and stored at −20°C until use. Every day the dose to inject was freshly prepared by diluting an aliquot in saline-Tween80 (1% final ethanol content, 5% Tween80) and then intraperitoneally administered. Body weight and food intake were monitored daily, and the amount of food ingested was converted to kilocalories. Food efficiency was calculated as body weight variation (in grams) over caloric intake (in Kcals). Five days before euthanasia all groups of mice were also daily injected with 75 mg/kg of 5-bromo-2′-deoxyuridine (BrdU) dissolved in saline (Sigma-Aldrich). At the end of the treatment period, mice were euthanized by cervical dislocation and tissues collected for further histological and biochemical analysis. Samples from adipose tissue, pancreas and liver were fixed in 4% PFA by overnight immersion and then paraffin-embedded for histochemistry and immunohistochemistry. In parallel, plasma, adipose, pancreatic and hepatic samples were also snap-frozen and stored at −80°C for biochemistry analysis. The number of animals used for each experiment is detailed in figure legends.

### Adipokines and Cytokines Determination

Plasma was obtained by centrifuging (2,000 × g, 4°C, for 10 min) whole blood in EDTA-coated tubes. Plasma levels of insulin and several inflammatory cytokines (IFN-γ, IL-5, IL-6, CXCL1, IL-10) were measured using the Meso Scale Discovery Multi array (Meso Scale Diagnostics, Rockville, MA, USA) in an MSD instrument (SECTOR S 600) equipped with multi-array electrochemiluminescence detection technology (Meso Scale Diagnostics). Plasma levels of leptin (BioVendor, Czech Republic) and HMW Adiponectin (Shibayagi Co., Japan) were measured by ELISA assay in accordance with manufacturer's instructions.

### Biochemical Analysis in Plasma Samples

The plasma lipid profile (Triglycerides, total cholesterol, HDL-c, LDL-c, NEFA, and glycerol), blood glucose level and the liver marker alanine transaminase (ALT) were determined by routine laboratory methods using a Cobas Mira autoanalyzer (Roche Diagnostic System, Basel, Switzerland) and reagents from Spinreact (Spinreact S.A.U., Girona, Spain) and Biosystems (Biosystems S.A., Barcelona, Spain). Plasma insulin levels were measured using a commercial ELISA assay according to manufacturer's instructions (Mercodia, Uppsala, Sweden).

### Lipid Peroxidation Measurement

Lipid peroxidation was determined by measuring thiobarbituric acid-reactive substances (TBARS). Malondialdehyde (MDA), a natural bi-product of lipid peroxidation reacts with thiobarbituric acid (TBA) to generate a MDA-TBA adduct that can be easily quantified at 532 nm in a spectrophotometer. Tissue samples were homogenized with ice-cold Tris-HCl buffer (150 mM KCl, 50 mM Tris, pH 7.4) supplemented with butylated hydroxytoluene to avoid artificial peroxidation during the test. The supernatant was incubated with MDA to obtain the TBARS. The absorbance was measured (VERSAmax, Molecular Devices LLC, San Jose, CA, USA) and interpolated in a standard curve using malondialdehyde-bisdiethyl-acetal (MDA, Sigma-Aldrich). The final values were expressed as nanomoles of TBARS per milligram of tissue.

### Glucose-Stimulated Insulin Secretion (GSIS)

Islets of Langerhans were isolated from two mice in each group by using the collagenase digestion method, as previously described (24). Islets were then cultured for 20–24 h in RPMI-1640 medium supplemented with 11 mM glucose (Invitrogen, CA, USA), 2 mM glutamine, 200 IU/ml penicillin, 200 μg/ml streptomycin and 8% fetal bovine serum stripped with charcoal-dextran (Invitrogen). For GSIS experiments islets were pre-incubated at 37 °C for 2 h in Krebs-bicarbonate buffer solution containing 14 mM NaCl, 0.45 mM KCl, 0.25 mM CaCl_2_, 0.1 mM MgCl_2_, 2 mM HEPES and 3 mM glucose, and equilibrated with 95% O_2_: 5% CO_2_ at pH 7.4. Size-matched islets, five in each well from a 24-well plate, were seeded in 0.5 mL fresh buffer containing 3 mM glucose or 11 mM glucose. Then islets were incubated for 1 h at 37°C, 5% CO_2_. After incubation, 1% bovine albumin was added to each well, and the plate was cooled at 4°C for 15 min to stop insulin secretion. Media were then collected and stored at −20°C until insulin measurement by ELISA (Mercodia, Uppsala, Sweden), according to the manufacturer's instructions.

### Islet Morphometric Analysis

Number of islets and islet area were assessed by morphometric analysis in pancreatic sections. Paraffin-embedded pancreases from each mouse were cut at four different levels and stained with haematoxylin and eosin. Low-magnification photomicrographs were taken in an Olympus BX41 microscope (Olympus Corporation, Tokyo, Japan) and analyzed by the ImageJ software.

### Liver Glycogen Measurement

Determination of hepatic glycogen was performed according to previous reports with some modifications (25, 26). Briefly, the liver samples (300–500 mg) were transferred to test tubes containing 30% KOH (w/v) and boiled for 1 h until complete homogenization. Na_2_SO_4_ was then added, and the glycogen was precipitated with ethanol. The samples were centrifuged at 800 g for 10 min, the supernatants were discarded, and the glycogen was dissolved in hot distilled water. Ethanol was added and the pellets obtained after a second centrifugation were dissolved in distilled water in a final volume of 25 ml. Glycogen content was measured by treating a fixed volume of sample with phenol reagent and H_2_SO_4_. Absorbance was then read at 490 nm with a spectrophotometer (VERSAmax, Molecular Devices LLC).

### RNA Isolation and Real Time PCR

Tissues (100 mg of adipose tissue) were dissected and mRNA isolated using Trizol Reagent (Sigma-Aldrich) and RNeasy Mini Kit (Qiagen) following manufacturer's instructions. Retrotranscription was performed using SuperScript IV RT (Thermo Fisher Scientific Inc., Waltman, MA, USA) and mRNA expression were analyzed in an Applied Biosystems^®^ 7500 fast using Fast Advanced Master Mix (all from Thermo Fisher Scientific) and appropriate FAM-labeled Taqman primers and probes for *Cxcl1, Il10*, and *Ucp1*. VIC-labeled primers and probe were used for housekeeping genes.

### Oil Red O Staining of Liver Ectopic Lipid Deposition

Frozen liver samples were sliced in a cryostat, attached to microscope slides, and air-dried at room temperature for 30 min. Liver sections were then stained in fresh Oil red O for 10 min, rinsed in distilled water and immediately counterstained with haematoxylin for 1 min. Photomicrographs were taken on an Olympus BX41 microscope and the Oil red O staining intensity was quantified by using Image J software.

### Masson's Trichrome Staining of Liver Fibrosis

Liver fibrosis was evaluated at the histological level by staining collagen fibers with Masson's trichrome staining. For that purpose, paraffin-embedded sections were hydrated and stained by using the Masson's trichrome kit (Casa Álvarez Material Científico S.A., Madrid, Spain) according to manufacturer's instructions.

### Immunohistochemistry and Immunofluorescence Staining

Paraffin-embedded tissue sections (3 μm) were dewaxed, hydrated, and treated with antigen unmasking solution, citric acid based (Vector Laboratories Inc., Burlingame, CA, USA) for 20 min in a steamer and then 20 min to cool down. Sections were washed thrice with phosphate buffered saline (PBS). Endogenous peroxidase was quenched with 2% H_2_O_2_ in PBS for 30 min with agitation and endogenous biotin, biotin receptors, and avidin binding sites were blocked by avidin/biotin blocking kit according to manufacturer's instructions (Vector Laboratories Inc.). Immunohistochemistry was performed by incubating overnight at 4°C with primary antibody 1/100 (anti-insulin, Sigma-Aldrich; anti-F4/80, Abcam, Paris, France; anti-pNFKB, Abcam), rinsed thrice with PBS, followed by HRP polymer-conjugated Goat anti-Rat/Mouse polyclonal antibody (1 h) and finally rinsed thrice again and developed with diaminobenzidine substrate. Slides were rinsed in tap water, lightly counterstained with Mayer's haematoxylin, rinsed in ammonium chloride dehydrated and mounted with DPX medium (Shandon, Pittsburgh, Pennsylvania, USA). Specific primary antibodies were substituted with PBS or non-immune isotype-matched sera as the negative control.

For immunofluorescence primary antibody (anti-insulin 1/100 overnight, Santa Cruz Biotechnology Inc., Dallas TX, USA; anti-5-bromo-2′-deoxyuridine 1/100 overnight, Sigma-Aldrich; anti-αSMA, 1/100 overnight, Santa Cruz Biotechnology) incubation was followed by anti-rabbit IgG-AlexaFluor488 and/or anti-mouse IgG-AlexaFluor568 (1/1000; Thermo Fisher Scientific), for 1 h at room temperature. Slides were coverslipped and protected from photobleaching by Fluoroshield Mounting Media (Sigma-Aldrich). Photomicrographs were taken on an Olympus BX41 and further processed by Image J software to quantify signal intensity.

### Apoptosis Assessment

Apoptosis was evaluated by the TUNEL technique using the *in situ* apoptosis detection kit (Roche) according to manufacturer's instructions. Images were analyzed using ImageJ software. Number of TUNEL-positive cells were normalized to islet area.

### Data Analysis

Data are expressed as mean ± standard error of the mean (S.E.M.) for data fitting a normal distribution and median ± min to max for non-normal distributions. The statistical significance of differences in mean or median values was assessed by Student *t*-test or analysis of variance (ANOVA) followed by Tukey's multiple comparison test for normal distributions and by Mann-Whitney test or Kruskal-Wallis test followed by Dunn's multiple comparison test for non-normal distributions. All analyses were performed with GraphPad Prism 6.07 or 7.04 (GraphPad Software, San Diego, CA, USA). A *p* < 0.05 was considered significant.

## Results

### ABN-CBD Does Not Affect Body Weight

Ten-week-old male C57Bl/6J mice were fed a high fat diet (HFD) or standard diet (SD) for 15 weeks. Mice fed HFD weighed significantly more than SD-fed mice (37.9 ± 0.9 vs. 32.6 ± 1.2 g respectively; Figure 1B). Diet induced obese (DIO) mice were glucose intolerant and insulin resistant but they did not show impaired fasting glucose [103 ± 7 vs. 108 ± 4 mg/dl, SD- and HFD-mice, respectively (22)]. DIO mice were then randomized to daily intraperitoneal injections of vehicle (HFD-vehicle mice) or 0.05 mg/kg of Abn-CBD (HFD-Abn-CBD mice) for 2 weeks (Figure 1A). Dosage for Abn-CBD was selected based on an ipGTT on lean mice (Supplementary Figure 1) and previous literature (15). After 2 weeks of treatment, body weights of HFD-vehicle and HFD-Abn-CBD mice were comparable (36.5 ± 0.8 and 38.9 ± 1.7 g, respectively), while SD-fed mice weighed 29.8 ± 1.8 g (Figures 1B,C). Both HFD-Abn-CBD and HFD-vehicle mice remained glucose intolerant (Supplementary Figure 2). Calorie intake per mouse was significantly higher in the HFD-Abn-CBD than in the HFD-vehicle group (Figure 1D), although calorie per gram (Figure 1E) and food efficiency was comparable in both groups (Figure 1F). In obesity, adiposity positively correlates with leptin plasma levels. HFD-vehicle mice had a 2.8-fold increase in plasma leptin levels compared to lean mice (Figure 1G). In agreement with their increased body weight and food consumption (Figures 1C,D), HFD-Abn-CBD mice had greater leptin levels (4.2-fold higher compared to lean mice) although differences found between HFD-vehicle and -Abn-CBD mice did not reach statistical significance (*p* = 0.07) (Figure 1G). Adiponectin, a hormone secreted from the adipose tissue to regulate glucose homeostasis, is known to be reduced in obesity (27). Plasma adiponectin levels were significantly lower in HFD-fed mice compared to SD-fed mice, independently of treatment (Figure 1H).

### Abn-CBD Does Not Alter the Plasma Lipid Profile

As DIO is associated with elevated triglycerides, free-fatty acids and cholesterol in circulation, we therefore analyzed the plasma lipid profile of SD-fed, HFD-vehicle, and HFD-Abn-CBD mice. Although plasma triglyceride levels in HFD-fed mice were similar to those found in SD-fed mice, total cholesterol was 1.3-fold higher in HFD-fed compared to SD-fed mice (Table 1). High density lipoprotein (HDL) and non-esterified fatty acid (NEFA) content in plasma were also significantly higher in HFD-fed mice than in plasma from SD-fed mice (1.5- and 1.6-fold, respectively; Table 1). There were no significant differences in low density lipoprotein (LDL) levels in plasma, neither of glycerol levels between HFD-fed and SD-fed mice (Table 1). The lipid profile found in plasma from HFD-Abn-CBD mice was comparable to the one found in HFD-vehicle mice, including high cholesterol, elevated HDL and NEFA (1.25-, 1.5- and 1.6-fold higher, respectively, than in SD-fed mice; Table 1).

**Table 1:** Systemic lipid markers.

**Systemic lipid markers**	**SD**	**HFD-vehicle**		**HFD-Abn-CBD**	
Triglycerides (mg/dl)	104.6 ± 7.9	103.4 ± 9.5	n.s.	112.4 ± 9.96	n.s.
Total cholesterol (mg/dl)	136.4 ± 10.7	174.8 ± 12.6^*^	^*^	170.3 ± 13.8	^*^
HDL-c (mg/dl)	42.4 ± 3.3	64.0 ± 4.1	^**^	65.1 ± 4.6	^**^
LDL-c (mg/dl)	14.7 ± 1.4	16.8 ± 2.82	n.s.	16.8 ± 2.5	n.s.
NEFA (mM)	2.1 ± 0.3	3.5 ± 0.1	^*^	3.6 ± 0.1	^*^
Glycerol (mg/l)	5.7 ± 2.9	10.0 ± 4.4	n.s.	12.9 ± 2.8	n.s.

* p ≤ 0.05 and

** *p ≤ 0.01 compared to SD-vehicle*.

*n.s., non-significant*.

### ABN-CBD Ameliorates Hyperinsulinemia, Protects Beta Cells From DIO-Induced Apoptosis, Decreases Inflammation, and Stimulates Beta Cell Proliferation

**Figure 2:**
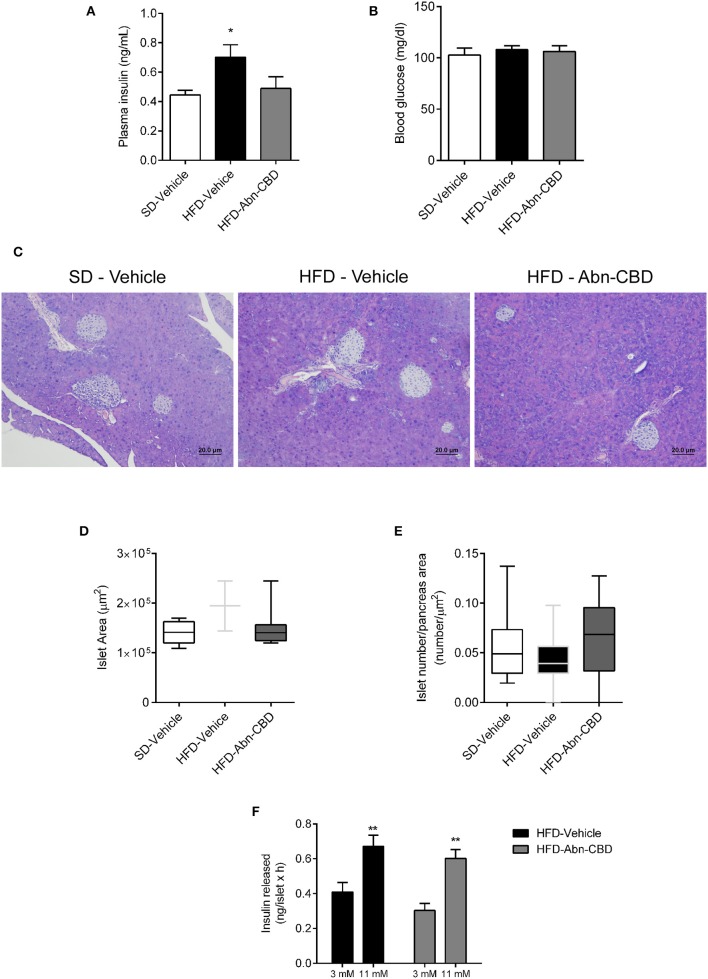
Effect of Abn-CBD on glucose homeostasis, islet morphology and functionality. **(A)** Fasted (overnight) plasma insulin and **(B)** blood glucose levels at the end of the study. **(C)** Representative photomicrographs of islets. **(D,E)** Morphometric analysis of islets. Data show mean ± S.E.M. except **(D,E)** that show median ± min to max. *n* = 6 SD-Vehicle, *n* = 7 HFD-Vehicle and *n* = 8 HFD-Abn-CBD except **(D)**, *n* = 51–66 islets from 4 mice each group, **(E)** *n* = 52–81 islets from 4 mice each group. ^*^p ≤ 0.05 and ^**^p ≤ 0.01 compared to SD-Vehicle. **(F)** Static glucose-stimulated insulin secretion in HFD-Vehicle and HFD-Abn-CBD isolated islets. *N* = 11–12 wells each condition, islets from 2 mice each group.

**Figure 3:**
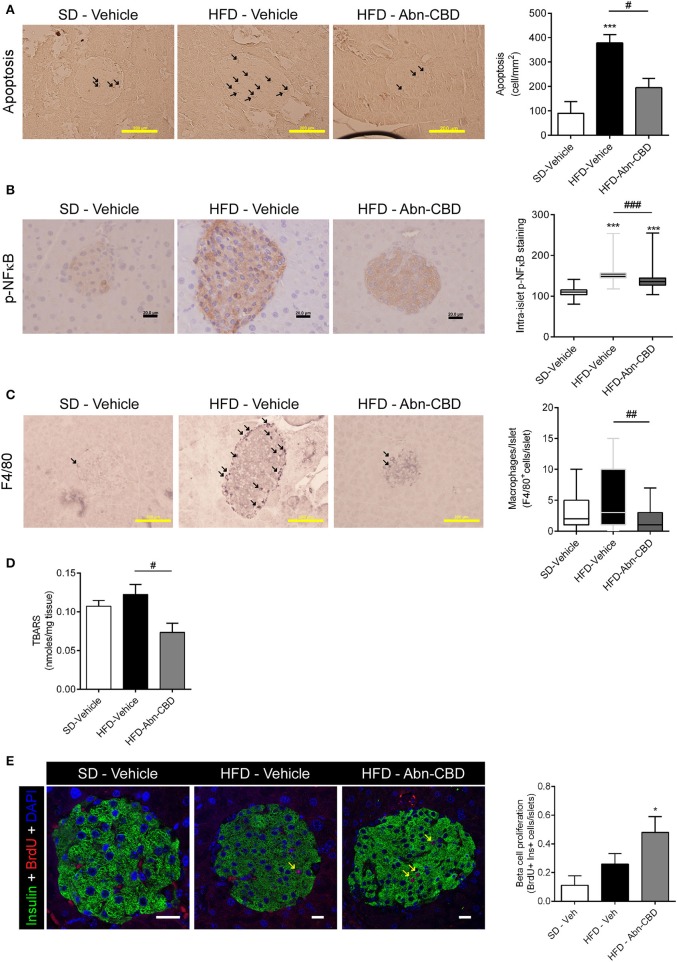
Effect of Abn-CBD on pancreatic beta-cell viability and intra-islet inflammation. Representative photomicrographs and quantification of islets immunostained for apoptosis **(A)** and p-NFκB **(B)**; arrows indicate apoptotic cells; p-NFκB immunostaining was counterstained with haematoxylin. **(C)** F4/80 staining; arrows indicate macrophages. **(D)** Quantification of lipid peroxidation by TBARS production. **(E)** Representative photomicrographs of double insulin (green) and BrdU (red) immunostaining in islets and quantification of BrdU+/Insulin+ cells; arrows indicate proliferative beta cells; scale bar is 20 μm. *n* = 6 SD-Vehicle, *n* = 7 HFD-Vehicle and *n* = 8 HFD-Abn-CBD. ^*^p ≤ 0.05 and ^***^p ≤ 0.001 compared to SD-Vehicle; ^#^*p* ≤ 0.05, ^*##*^*p* ≤ 0.01, and ^###^*p* ≤ 0.001 compared to HFD-Vehicle.

After 2 weeks of treatment with vehicle or Abn-CBD, HFD-vehicle mice were hyperinsulinemic compared to SD-fed mice (Figure 2A) while blood glucose levels were comparable (Figure 2B). These data suggest that mice remained insulin resistant as before starting the treatment but had not yet developed diabetes. Remarkably, mice treated with Abn-CBD had a similar insulinemia than SD-vehicle (Figure 2A) while maintaining blood glucose at the same level as SD- and HFD-vehicle mice (Figure 2B). We analyzed the pancreatic islets of Langerhans both at the morphometric and functional level (Figures 2C–F). No changes in number of islets were detected among groups (Figure 2E). However, mice fed a HFD showed a strong tendency toward larger islets than SD-fed mice (*p* = 0.06) while this hypertrophy was absent in HFD-Abn-CBD islets (Figure 2D). Treatment with Abn-CBD had no effect on total insulin secretory capacity, as shown by a static *in vitro* glucose-stimulated insulin secretion assay (Figure 2F). In DIO, loss of beta cell mass is associated with increased inflammation and oxidative stress in beta cells that leads to beta cell death. Accordingly, we found increased intra-islet apoptosis (Figure 3A), phosphorylation of p65 (p-NFκB) (Figure 3B) and a slight non-significant increase in pancreatic TBARS (Figure 3D) in HFD-vehicle when compared to SD-Vehicle. Importantly, Abn-CBD significantly reduced pancreatic content of TBARS (Figure 3D), intra-islet pNFκB staining (Figure 3B) and macrophage infiltration (Figure 3C), greatly lessening apoptosis (Figure 3A). Moreover, Abn-CBD induced beta cell proliferation, as measured by BrdU positive staining of beta cells, compared to SD-fed mice (Figure 3E). Thus, Abn-CBD protected beta cell mass without altering beta cell function.

### Abn-CBD Reduces DIO-Induced Meta-Inflammation

**Figure 4:**
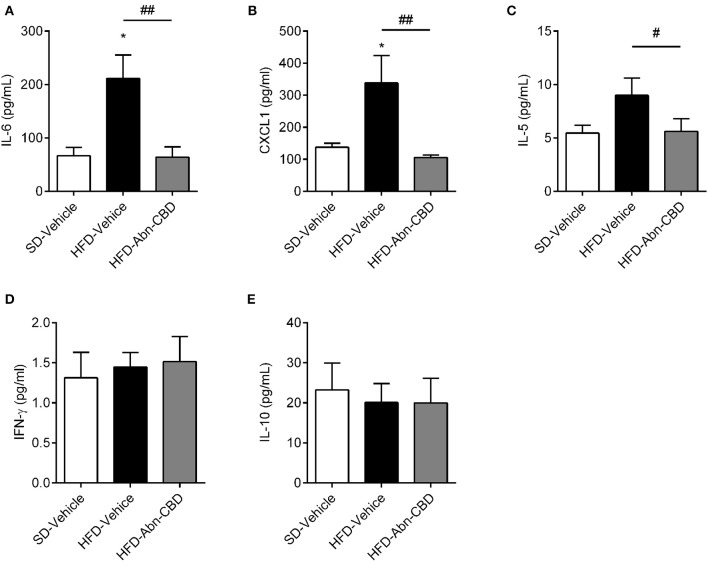
Effect of Abn-CBD on circulating inflammatory cytokines. Plasma levels of IL-6 **(A)**, CXCL1 **(B)**, IL-5 **(C)**, IFN-γ **(D)**, and IL-10 **(E)** after the 2-week treatment of mice. *n* = 6 SD-Vehicle, *n* = 7 HFD-Vehicle and *n* = 8 HFD-Abn-CBD. ^*^p ≤ 0.05, compared to SD-Vehicle; ^#^*p* ≤ 0.05 and ^##^*p* ≤ 0.01 compared to HFD-Vehicle.

Low-grade chronic inflammation is associated with DIO-related complications. Accordingly, plasma of HFD-vehicle mice showed significantly higher levels of interleukin 6 (IL-6; 3.2-fold increase; Figure 4A) and CXCL-1 (2.4-fold increase; Figure 4B) compared to SD-fed mice, while interleukin 5 (IL-5) levels trended to be 1.6-fold higher (Figure 4C). Of importance, treatment with Abn-CBD restored the levels of IL-6, CXCL-1, and IL-5 to those found in SD-fed mice (Figures 4A–C). HFD-fed mice did not show any change in either interferon gamma (IFNγ; Figure 4D) nor interleukin 10 (IL-10; Figure 4E) compared to SD-fed mice, independently of the treatment.

**Figure 5:**
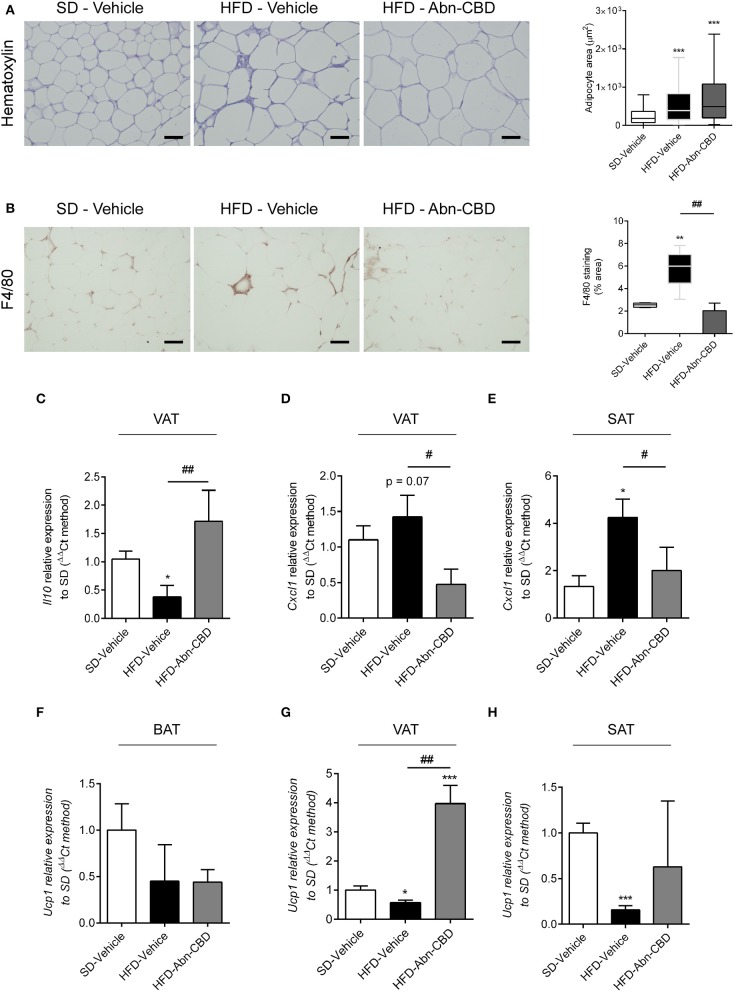
Effect of Abn-CBD on inflammation in white adipose tissue. Representative photomicrographs and quantification of **(A)** adipocyte size and **(B)** crown-like structures in white adipose tissue. Relative expression of **(C)** *Il10* and **(D)** *Cxcl1* in visceral adipose tissue and of **(E)** *Cxcl1* in subcutaneous adipose tissue. Relative expression of *Ucp-1* in **(F)** brown adipose tissue, **(G)** visceral adipose tissue, and **(H)** subcutaneous adipose tissue. β-actin was used as reference gene. ^*^p ≤ 0.05, ^**^p ≤ 0.01, and ^***^p ≤ 0.001 compared to SD-Vehicle; ^#^*p* ≤ 0.05 and ^##^*p* ≤ 0.01 compared to HFD-Vehicle.

Since obesity is strongly associated to inflammation at white adipose tissue (WAT) (28), we analyzed the inflammation occurring in visceral (VAT) and subcutaneous adipose tissue (SAT). HFD-fed mice showed an expected increase in adipocyte size compared to SD-fed mice, independently of treatment (Figure 5A). We found an increase in the presence of crown-like structures in HFD-vehicle WAT upon staining with F4/80, which was significantly decreased in WAT from Abn-CBD-treated mice (Figure 5B). Analysis of cytokine expression showed a significant reduction of *Il10* expression in VAT (Figure 5C), and a significant increase in the expression of *Cxcl1* in both VAT (*p* = 0.07) and SAT from HFD-vehicle mice (Figures 5D,E, respectively). Interestingly, treatment with Abn-CBD prevented the alterations observed in cytokine expression in VAT (Figures 5C,D) and SAT (Figure 5E), showing that Abn-CBD protects WAT from diet-induced inflammation. We also wanted to explore whether Abn-CBD could promote a shift toward browning in WAT. For this purpose we analyzed *Ucp1* expression in BAT, VAT, and SAT. While no significant differences were found in brown adipose tissue among groups (Figure 5F), HFD-vehicle mice showed a significant decrease in *Ucp1* expression in VAT and SAT that was counteracted by Abn-CBD (Figures 5G,H).

### Abn-CBD Does Not Protect Against NAFLD

**Figure 6:**
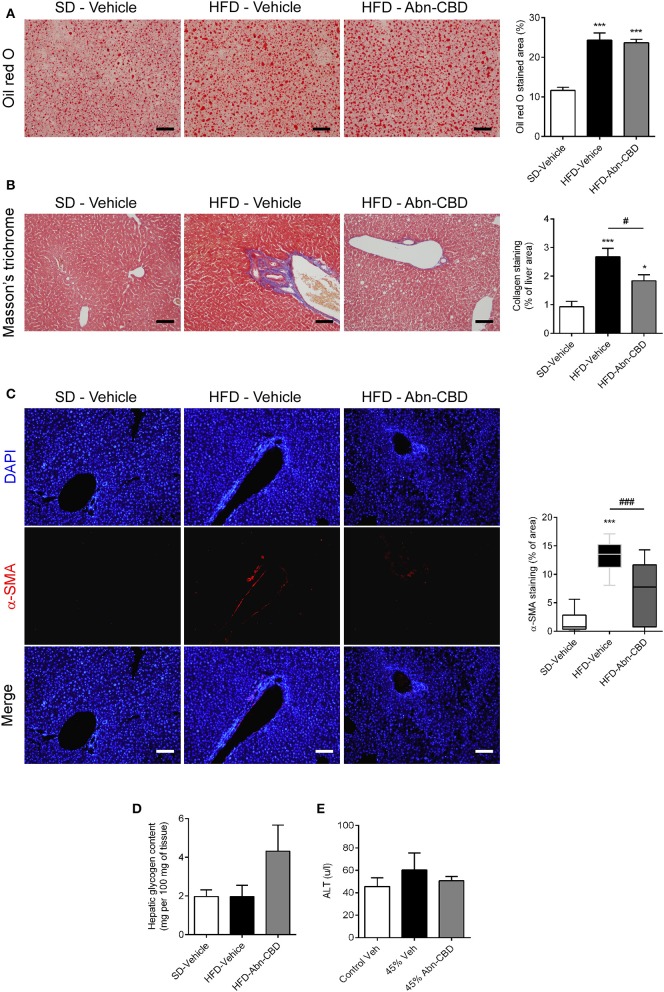
Effect of Abn-CBD on liver structure and function. Representative photomicrographs and quantification of livers stained for lipid droplets **(A)** and collagen fibers **(B)**, scale bar is 100 μm. Representative photomicrographs of immunofluorescence in liver for α-SMA (red), counterstained with DAPI (in blue) **(C)**. Biochemical determination of hepatic glycogen content **(D)** and circulating levels of alanine aminotransferase **(E)**. *n* = 7–8 SD-Vehicle, *n* = 5–8 HFD-Vehicle and *n* = 6–8 HFD-Abn-CBD. ^*^p ≤ 0.05 and ^***^p ≤ 0.001 compared to SD-Vehicle; ^#^*p* ≤ 0.05 and ^###^*p* ≤ 0.001 compared to HFD-Vehicle.

In the liver the accumulation of triglycerides leads to NAFLD that eventually leads to liver failure (9). Herein, DIO mice accumulated triglycerides in liver independently of treatment, displaying 2-fold increase in lipid content compared to lean mice, as shown by oil red-O staining (Figure 6A). Furthermore, liver from HFD-vehicle mice exhibited fibrosis, as determined by a significant increase in collagen content compared to SD-fed mice (Figure 6B) as well as early makers of liver fibrosis, such as α-SMA (Figure 6C). Interestingly, the liver from HFD-Abn-CBD mice had significantly lower collagen than HFD-vehicle mice (Figure 6B) and reduced staining of α-SMA (Figure 6C). Moreover, liver glycogen content was comparable in both HFD- and SD-fed vehicle-treated mice (Figure 6D). Although not reaching statistical significance (*p* = 0.07), HFD-Abn-CBD mice displayed a 2.2-fold higher content of hepatic glycogen than HFD-vehicle mice (Figure 6D). In order to assess liver damage, we measured plasma levels of alanine aminotransferase (ALT). Levels of ALT in DIO mice were comparable to those found in lean mice, independently of treatment (Figure 6E).

### Abn-CBD Protects Hepatocytes From DIO-Induced Immune Cell Infiltration

**Figure 7:**
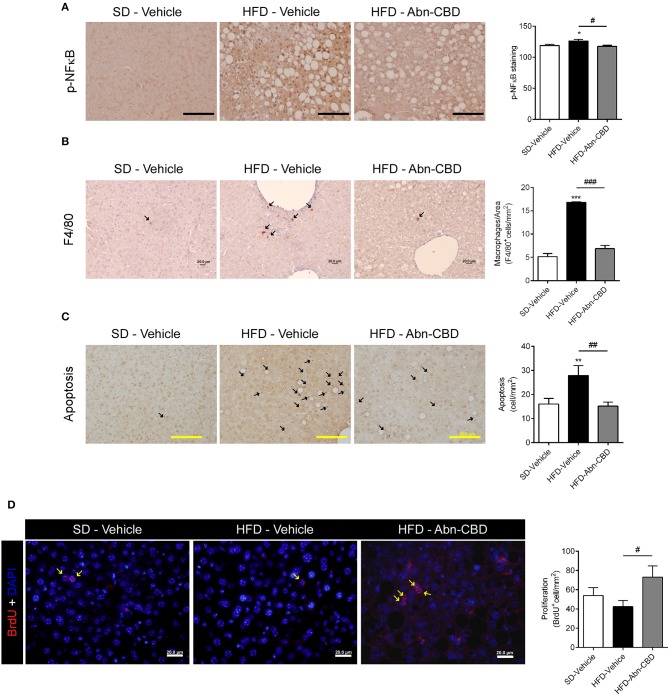
Effect of Abn-CBD on hepatocyte viability and liver inflammation. **(A)** Representative photomicrographs and quantification of livers stained for the inflammatory marker p-NFκB **(A)**, the macrophage marker F4/80 **(B)** and apoptotic cells **(C)**; arrows indicate stained cells; scale bar is 100 μm **(A)**, 20 μm **(B)**, and 200 μm **(C)**. **(D)** Representative photomicrographs and quantification of BrdU immunofluorescence (in red) in the liver, counterstained with DAPI (in blue). Arrows indicate proliferating cells. *n* = 7–8 SD-Vehicle, *n* = 5–8 HFD-Vehicle and *n* = 6–8 HFD-Abn-CBD. ^*^p ≤ 0.05, ^**^p ≤ 0.01, and ^***^p ≤ 0.001 compared to SD-Vehicle; ^#^*p* ≤ 0.05, ^##^*p* ≤ 0.01, and ^###^*p* ≤ 0.001 compared to HFD-Vehicle.

As HFD-Abn-CBD mice displayed reduced liver fibrosis concomitant with lower levels of several circulating pro-inflammatory cytokines compared to HFD-vehicle mice, we further assessed the degree of liver inflammation. Liver from HFD-vehicle mice had increased p-NFκB compared to liver from SD-fed mice, while liver from HFD-Abn-CBD mice had significantly reduced p-NFκB levels comparable to those in liver from SD-fed mice (Figure 7A). Additionally, liver from HFD-vehicle mice showed a significant 3.3-fold increase in the number of macrophages (F4/80^+^ cells) compared to SD-fed mice (Figure 7B). Treatment with Abn-CBD greatly reduced the number of macrophages to the levels found in SD-fed mice (Figure 7B). We then determined hepatocytes viability measured as apoptosis and proliferation. Hepatocytes from HFD-vehicle mice were undergoing significantly more apoptosis than those from SD-fed mice, which was reverted upon Abn-CBD treatment (Figure 7C). In addition, Abn-CBD increased hepatocytes proliferation when compared to HFD-vehicle, but not over those levels found in hepatocytes from SD-fed mice (Figure 7D).

## Discussion

CBD and Abn-CBD lack psychoactive effects, and therefore bestow them as attractive candidates in medicinal cannabis research. Here, we investigated the *in vivo* actions of Abn-CBD in the metabolic and inflammatory dysfunctions that arise in the context of obesity, prediabetes, and NAFLD. At the biochemical and cellular level, C57Bl6/J mice fed a 45% HFD (saturated fats from lard) for 15 weeks displayed, dyslipidaemia, hyperleptinemia, hypoadiponectinemia, hyperinsulinemia, and increased islet cell apoptosis. In addition, mice developed fatty liver with macrophage infiltration, increased hepatocyte apoptosis and decreased hepatocyte proliferation. However, we did not detect impaired liver function, underlining the validity of this mouse model to assess new therapies tackling early stages of obesity comorbidities.

Overall, subchronic treatment with Abn-CBD in this mouse model improved low-grade inflammation, reverted hyperinsulinemia and decreased liver fibrosis without altering body weight or ectopic accumulation of fat in the liver.

Although Abn-CBD did not impact either body weight or the lipid profile, it increased leptin levels. CBD has been described to decrease food intake in acute tests (29, 30) but its impact on body weight is poorly investigated and controversial (31, 32). Previous data using a streptozotocin (STZ)-induced diabetic mouse model showed that Abn-CBD lowered food intake (15), which was associated with restored plasma insulin levels. The discrepancies of our results with these findings may lay on the obvious differences between STZ-model and DIO-model. Indeed STZ-model does not induce obesity, insulin resistance and hyperinsulinemia, suggesting that underlying mechanisms for Abn-CBD-driven changes in food intake are different in insulin resistant model and in the insulin production deficient model.

Inflammation and insulin resistance are intimately related processes, and currently there are evidence suggesting that inflammation is driven by insulin resistance (33). Unfortunately, we could not measure insulin tolerance or insulin sensitivity after Abn-CBD treatment, but the absence of hyperinsulinemia without increase in fasting glucose levels indirectly suggest that Abn-CBD might improve insulin sensitivity in our mouse model. However, gold-standard techniques such as euglycemic hyperinsulinemic clamp would be necessary in order to unequivocally establish the impact of Abn-CBD in insulin tolerance/sensitivity. Given that direct anti-inflammatory properties have been ascribed to Abn-CBD and other GPR55 agonists (19, 34, 35), decreased inflammation in our model could be related to improved insulin sensitivity, direct actions on receptors such as GPR18 and GPR55, or both. Further studies in this regard would be required to decipher the molecular mechanism that underlies the role of Abn-CBD in insulin sensitivity toward inflammatory processes.

We and others have previously demonstrated that Abn-CBD can directly stimulate insulin secretion *in vitro* (21, 23) as well as *in vivo* in a mouse model of mild type 1 diabetes (15). However, to the best of our knowledge it has not been assayed in a mouse model of obesity and prediabetes. Herein, Abn-CBD did not seem to interfere with islet function as it did not change islet sensitivity to glucose as assessed by GSIS *ex vivo* experiments. Although subchronic Abn-CBD treatment in prediabetes does not enhance insulin secretion, we report here that hyperinsulinemia as well as the trend to increase islet area in HFD-vehicle mice were reverted, suggesting that subchronic Abn-CBD treatment in prediabetes reduces the inflammatory state that leads to beta cell death.

Notwithstanding, immunohistological analysis of pancreas showed that treatment with Abn-CBD greatly lowered islet cell apoptosis while induced beta cell proliferation, thus promoting preservation of beta cell mass. These findings are in agreement with our previous work showing a proliferative and protective role of Abn-CBD against cytokine-induced apoptosis on isolated islets from lean mice and human (21). Similar observations were also obtained in STZ-induced diabetic mice by another group (15). Importantly, cannabinoids and cannabinoid receptors have been previously shown to modulate islet viability in DIO mice (36, 37). Herein, we also found a significant reduction in oxidative stress in pancreas isolated from HFD-Abn-CBD mice compared to HFD-vehicle mice. Moreover, Abn-CBD significantly lowered levels of phosphorylated (i.e., active) NFκB corroborating with lower cell apoptosis in islets from HFD-Abn-CBD treated mice. Thus, Abn-CBD preserves islet viability and beta cell mass *in vivo* by reducing islet cell apoptosis and enhancing beta cell proliferation.

Importantly, the pro-adiposity effect found with Abn-CBD treatment was not only devoid of an extra pro-inflammatory component but rather accompanied by decreases in plasma levels of cytokines involved in systemic low-grade inflammation such as IL-6, IL-5, and CXCL1 levels. In fact, IL-6 is one of the primary mediators of low-grade inflammation in obesity (3) and both CXCL-1 as well as IL-5 have been found to be altered in prediabetes (6, 38). There are some evidence pointing at Abn-CBD as a modulator of the inflammatory response (19) and the two main G-protein coupled receptors (GPCRs) that so far have been related to Abn-CBD effects, i.e., GPR18 and GPR55, have been widely involved in inflammation and related processes (39, 40). Comparable to our data, the GPR55 agonist O-1602 significantly reduced the levels of IL-6 both in plasma and pancreas tissue in mice with cerulein-induced pancreatitis (34). Therefore, our results support an anti-inflammatory effect of Abn-CBD in prediabetes and NAFLD. The putative involvement of Abn-CBD in browning processes has not been explored so far. Our results show that Abn-CBD potently increases *Ucp1* expression in VAT, also counteracting HFD-induced decrease in SAT. This is suggestive of Abn-CBD promoting browning in WAT, what potentially could be contributing to decreased meta-inflammation. This first evidence warrants further investigation on the role that Abn-CBD may have on energy expenditure. NAFLD is an increasing concern in obesity-related comorbidities as it worsens metabolic syndrome, development of insulin resistance and cardiovascular disease (41). As expected, HFD induced a prominent increase of ectopic fat in the liver of our mouse model. Although Abn-CBD treatment did not resorb fat depot, it decreased collagen staining, suggesting a reduce fibrosis, most probably by reducing the pro-inflammatory environment. Importantly, Abn-CBD treatment did not affect liver function since both its capacity to store glycogen and the marker of liver function ALT were not compromised. Previously, CBD has been found to protect the liver from both alcohol-induced and non-alcohol-induced steatosis by mechanisms including inhibition of oxidative stress, increase in autophagy and decrease of lipid accumulation (42, 43). In contrast, a recent report found a deleterious effect of chronic administration of the atypical cannabinoids O-1918 and O-1602 in the liver of DIO Sprague-Dawley rats (44). However, administered doses were 100-fold higher than those used in our study, which could account for increased off-target effects and chemical toxicity. We also found that Abn-CBD decreased apoptosis and preserved proliferation in liver cells. An enhanced hepatocyte proliferation could carry the risk of developing hepatocellular carcinoma, which is related to the onset of NAFLD (45). In fact, high levels of IL-6 suppress hepatocyte proliferation in obesity (46), maybe as a protective mechanism. However, Abn-CBD increased hepatocyte proliferation only when compared to HFD-vehicle but proliferative levels were not above those found in healthy mice (SD-fed mice). This agrees with Abn-CBD preserving hepatocytes proliferation without inducing tumorogenesis. Interestingly, Abn-CBD has been reported to have anti-tumoral activity rather than promoting uncontrolled proliferation (47, 48). Given that Abn-CBD also decreased IL-6 levels in our mice, the beneficial effects of Abn-CBD on preserving hepatocytes proliferation might be mediated through reductions in IL-6 levels. The number of macrophages infiltrating the liver and activation of p-NFκB pathway was also diminished, suggesting a reduced intra-liver inflammation. Interestingly, IL-6, for which DIO-mediated increases were reverted by Abn-CBD in our study, has been found to play an important role in obesity-induced liver inflammation (49, 50). CBD was also shown to protect the liver by modulating inflammation, oxidative stress and cell death (51). Of note, CBD acts as a functional antagonist of CB1R (52), whose blockade was reported to revert liver steatosis in DIO mice (53). Moreover, O-1918, considered an antagonist of the Abn-CBD receptor, increased the levels of circulating pro-inflammatory cytokines in DIO rats (44). Taken together, these results point to Abn-CBD promoting a healthier cellular environment also in the liver.

In summary, we herein provide evidence that the atypical cannabinoid Abn-CBD is able to induce beneficial metabolic and anti-inflammatory actions at both systemic and tissue level in a mouse model of diet-induced prediabetes and NAFLD. Considering the sex limitation of our study -performed in males only-, further studies to confirm these effects in females are warranted. These results warrant further investigation on the potential this compound and/or related molecules may have to treat early stages of obesity-induced metabolic diseases.

## Data Availability Statement

All datasets generated for this study are included in the article/Supplementary Material.

## Ethics Statement

The animal study was reviewed and approved by Ethic Committee of the University of Malaga (authorization no. 2012–0061A).

## Author Contributions

SR-Z and FB-S conceived, designed, and supervised the study. SR-Z, IG-M, MG-F, VE-J, MP-M, AE-S, LS-S, EL, NC-V, and AR performed the experiments. SR-Z, GR-M, GO, BG, IG-M, and FB-S analyzed the results. FB-S, SR-Z, and IG-M wrote the manuscript. FB-S was the guarantor of this study.

### Conflict of Interest

The authors declare that the research was conducted in the absence of any commercial or financial relationships that could be construed as a potential conflict of interest.
