# Supplementary figures and images for: The Atypical Cannabinoid Abn-CBD Reduces Inflammation and Protects Liver, Pancreas, and Adipose Tissue in a Mouse Model of Prediabetes and Non-alcoholic Fatty Liver Disease
Source: Front Endocrinol (Lausanne). 2020 Mar 6;11:103. doi: 10.3389/fendo.2020.00103 (PMC7067697; doi:10.3389/fendo.2020.00103)

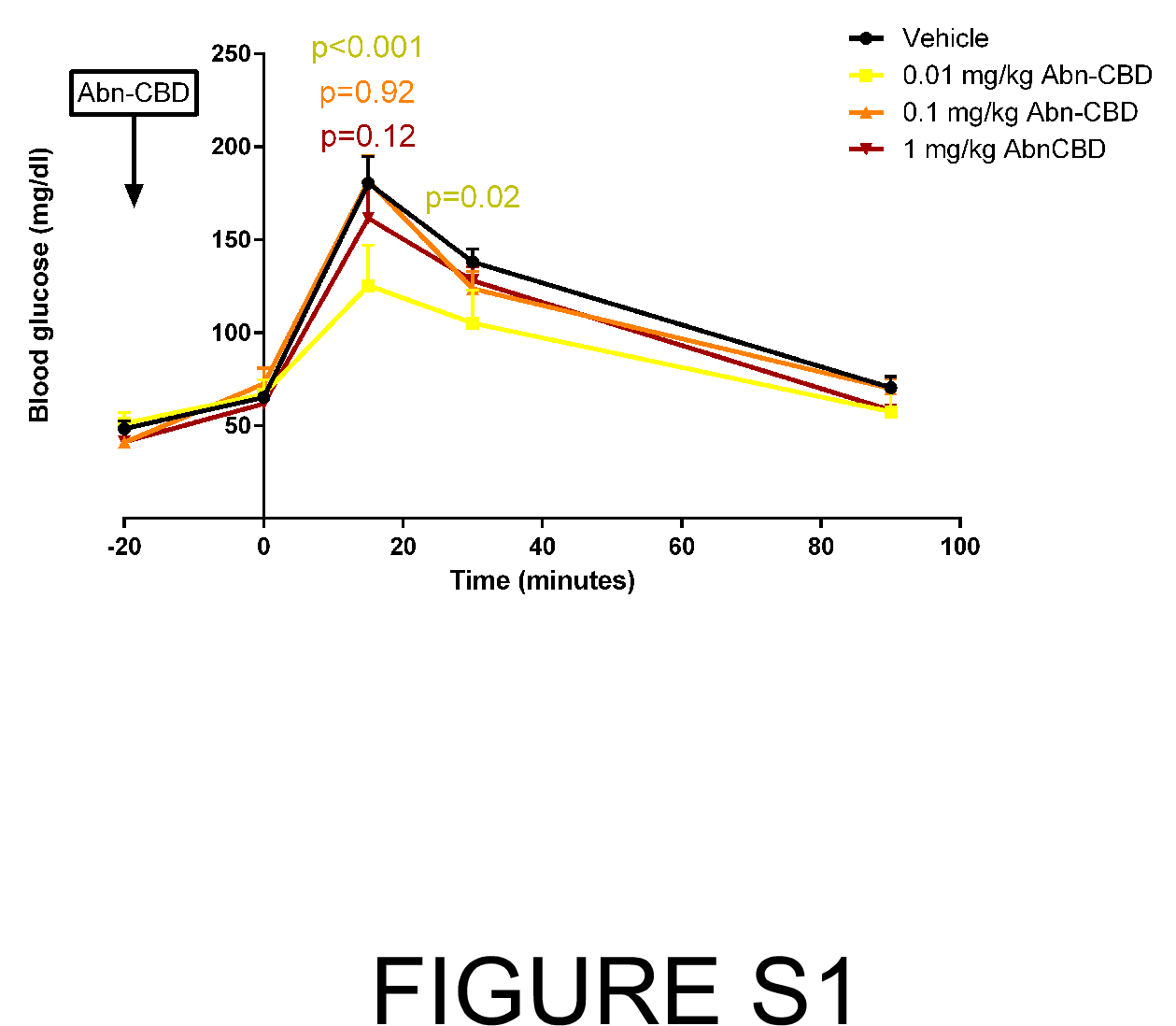

Supplement: Supplementary file 1 [file Image_1.TIF]

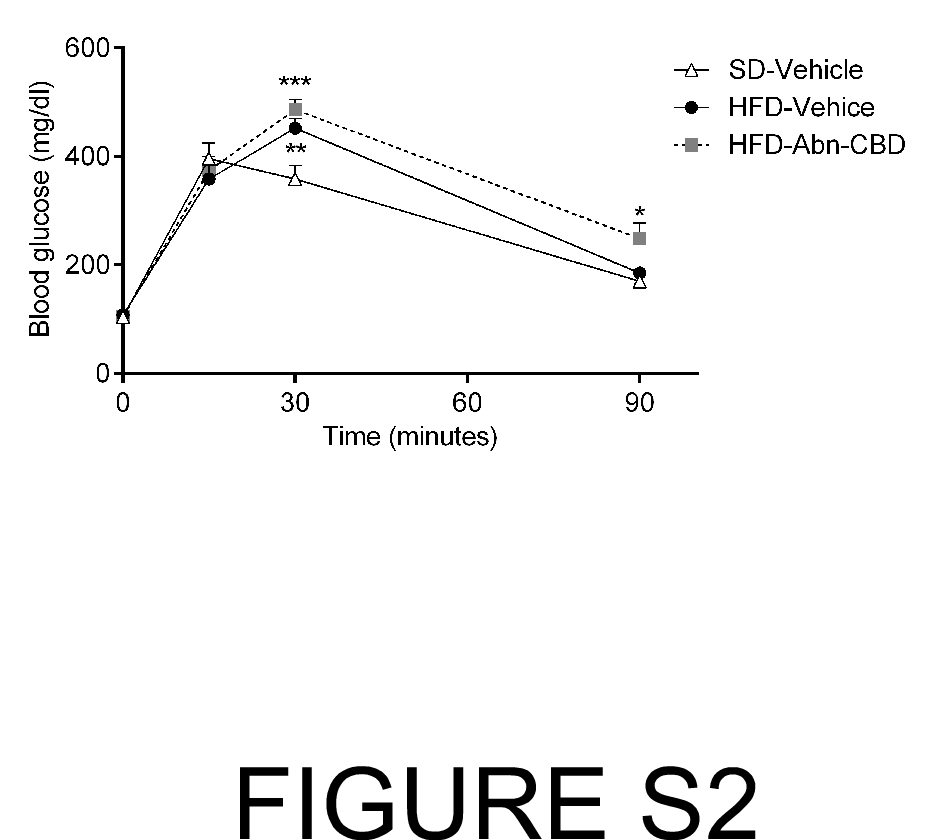

Supplement: Supplementary file 2 [file Image_2.TIF]
